# Predicting implementation of response to intervention in math using elastic net logistic regression

**DOI:** 10.3389/fpsyg.2024.1410396

**Published:** 2024-10-02

**Authors:** Qi Wang, Garret J. Hall, Qian Zhang, Sara Comella

**Affiliations:** Department of Educational Psychology and Learning Systems, College of Education, Health, and Human Sciences, Florida State University, Tallahassee, FL, United States

**Keywords:** math achievement, response-to-intervention, elastic net logistic regression, multiple imputation, random forest algorithm, variable selection

## Abstract

**Introduction:**

The primary objective of this study was to identify variables that significantly influence the implementation of math Response to Intervention (RTI) at the school level, utilizing the ECLS-K: 2011 dataset.

**Methods:**

Due to missing values in the original dataset, a Random Forest algorithm was employed for data imputation, generating a total of 10 imputed datasets. Elastic net logistic regression, combined with nested cross-validation, was applied to each imputed dataset, potentially resulting in 10 models with different variables. Variables for the models derived from the imputed datasets were selected using four methods, leading to four candidate models for final selection. These models were assessed based on their performance of prediction accuracy, culminating in the selection of the final model that outperformed the others.

**Results and discussion:**

Method_50_ and Method_coef_ emerged as the most effective, achieving a balanced accuracy of 0.852. The ultimate model selected relevant variables that effectively predicted RTI. The predictive accuracy of the final model was also demonstrated by the receiver operating characteristic (ROC) plot and the corresponding area under the curve (AUC) value, indicating its ability to accurately forecast math RTI implementation in schools for the following year.

## Introduction

Despite the well-known recommendations for practice regarding the use of response-to-intervention (RTI; often, but not always, synonymous with multi-tiered systems of support; Burns et al., 2016) to prevent and intervene on academic difficulties, there remains surprisingly little evidence on what factors help understand schools’ adoption of RTI in math. Although there is inconclusive evidence on the effectiveness of RTI as a prevention and intervention system (particularly in reading, e.g., Balu et al., 2015), RTI (and tiered intervention models in general) is well-established in the best practices for making data-based decisions on intervention need and preventing school-wide academic difficulties (Lane et al., 2019; McIntosh and Goodman, 2016; Schulte, 2016). Thus, it is necessary to better understand the conditions that predict schools’ adoption of RTI, especially in math, which has historically lagged reading in RTI research. In this study, we investigate the predictors of elementary schools’ adoption of math RTI using school-level data and school personnel surveys from a large, United States national dataset (Early Childhood Longitudinal Study: Kindergarten Cohort 2010-11; ECLS-K: 2011). Our study uses novel machine learning and cross-validation methods to identify the most prominent predictors of schools’ RTI adoption.

### What is RTI?

Response to intervention is a tiered model of intervention service delivery for academic skills. The prototypical RTI model encompasses three tiers of services, where Tier 1 (universal supports) refers to the general education curriculum that all students receive, Tier 2 refers to supplemental intensified interventions that support Tier 1 learning (e.g., small group reading interventions twice a week), and Tier 3 refers to intensive individualized and frequent (4–5 times a week) intervention designed to remediate substantial learning difficulties (Mellard et al., 2010). Together, RTI’s approach to implementation, prevention, and intervention is heavily rooted in public health prevention models (Schulte, 2016). RTI has been frequently contrasted with “wait-to-fail” models of service delivery where intensive intervention might not be provided until a student is referred for special education for a learning disability (though RTI has historically demonstrated its own shortcomings; Reynolds and Shaywitz, 2009). To this end, in addition to the multiple tiers of RTI providing a continuum of supports for all students, RTI also provides a mechanism to identify students that do not respond to the general supports available, which may open the potential for special education evaluation for a specific learning disability (Burns et al., 2016). RTI is typically applied in reading, math, and writing subject areas, and more recent conceptualizations of RTI integrate this approach with tiered social–emotional-behavioral supports (Lane et al., 2019; McIntosh and Goodman, 2016).

The nature of RTI warrants a school-wide implementation approach to build systems capacity for tiered service provision, including data collection (e.g., screening and progress monitoring), intervention material development/curation, implementation, and sustainment. Thus, while specific interventions *within* RTI may be implemented for small groups (e.g., Tier 2) or individuals (e.g., Tier 3), the overall model operates as a school-wide effort of data-based decision making based on screening and progress monitoring (including entry and exit criteria for intervention), intervention planning, development, and provision. Moreover, the public health approach that emphasizes population-wide “inoculation” mechanisms at tier 1 to increase the efficiency and efficacy of more intensive interventions intends to promote school effectiveness and achievement on a broad scale to mitigate the incidence of compounding academic difficulties (Mellard et al., 2010).

### Limitations of current math RTI implementation research

Despite the systems-level focus of RTI and the popularity of this model as a prevention system in K-12 schools, there is a dearth of research on the factors that relate to whether schools use RTI. Specifically, this is the case with math, as reading has traditionally predominated in schools’ RTI practices. Although there are some case studies and qualitative inquiry into the implementation of math RTI models (Bouck and Cosby, 2019; Donovan and Shepherd, 2013; Mason et al., 2019) as well as limited empirical investigation (Schumacher et al., 2017), the empirical research on factors predicting the use of RTI is lacking. This is a significant limitation in understanding the systematic factors that may determine whether schools choose to adopt an RTI model for prevention and intervention in math. The system capacity to adopt parallel RTI models for math may be limited if schools are already devoting substantial resources to other programs and initiatives (Mason et al., 2019). However, other contextual factors may play a part in the adoption of math RTI, including school resources, community contextual factors, and personnel factors (e.g., staff training background; Mason et al., 2019).

### Identifying predictors

With seemingly innumerable factors that could possibly relate to whether schools use math RTI, *a priori* selection of specific predictive factors may induce bias in predictions or unintentionally limit the scope of predictive factors. Compounding the identification of relevant predictors is the limited research base on school factors associated with math RTI implementation, aside from factors that would likely be related to the time and resources schools have available to conduct math RTI (e.g., economic conditions, community context, and student-related issues that guide service priorities). Consequently, narrowing the scope of predictors is an exploratory task at this stage.

Due to the large number of predictors and the relatively small sample size, elastic net logistic regression with nested cross-validation (nested CV) is a viable method to address the issues of predictor selection. This choice was motivated by the potential for overfitting when using traditional logistic regression (Hastie et al., 2006). The elastic net logistic regression can effectively address this risk by imposing penalties on the predictors, effectively reducing their number. This strategy not only alleviates concerns related to overfitting but also enhances the model’s predictive accuracy (Hans, 2011; Zou and Hastie, 2005). Therefore, determining the appropriate penalties is crucial when using elastic net regression. The most commonly used method to select these penalties is cross-validation, with *K*-fold cross-validation (CV) being particularly popular. However, Vabalas et al. (2019) showed that *K*-fold CV would inflate the accuracy of a regularized logistic regression with feature selection, especially when the sample size was small. Hence, they recommended using nested CV which could produce an accurate misclassification rate, even with a small sample size.

### The current study

**Figure 1:**
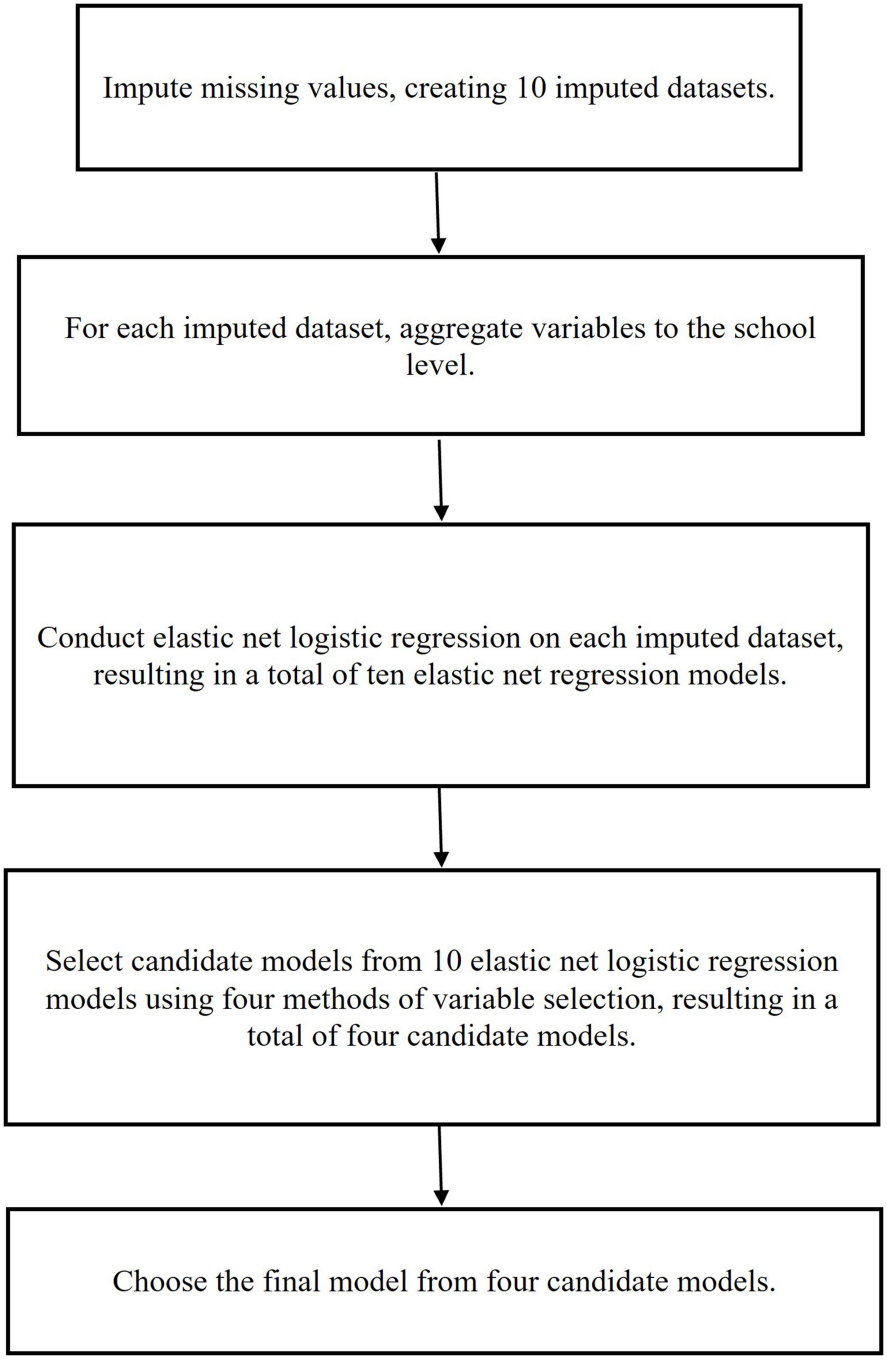
Data analysis procedure.

In this study, we used data from Grades 1 (2011–2012) and 2 (2012–2013) of the ECLS-K: 2011 to investigate the school-level factors in school year (SY) 2011–2012 that predicted elementary schools’ adoption of math RTI based on school administrator reports in SY2012-2013. Our primary research question was the following: What are the prominent predictors of schools’ math RTI implementation in the subsequent year? We hypothesized that factors related to school resources, achievement, and school personnel (e.g., training) would predominate as math RTI implementation predictors, as prior research has suggested that resource (e.g., staff training) and logistical factors (e.g., available time for implementation) are relevant to the adoption and implementation of math RTI (Choi et al., 2022; Mason et al., 2019). We used regularized regression techniques with nested CV to identify the most robust predictors. Due to missing values in this dataset, we initially used the Random Forest algorithm to create different imputed datasets. Subsequently, we applied elastic net logistic regression with nested CV to build prediction models for each imputed dataset. Since the penalties in elastic net logistic regression were evaluated separately in each dataset, this resulted in different penalties across the datasets, leading to variations in the logistic regression models. To address this, we employed four variable selection methods to identify the most robust predictors across the various elastic net logistic regression models from different imputed datasets, forming four candidate models. Then, four candidate models were evaluated using an independent dataset. The candidate model that exhibited the best performance among the four was selected as the final model. The flow chart of the data analysis procedure for this study is depicted in Figure 1.

## Methods

### Participating schools

The ECLS-K: 2011 is a multi-stage stratified nationally representative sample of United States students from kindergarten through fifth grade. The study initially samples base-year students in kindergarten, and students are followed through fifth grade with procedures to ensure the national representativeness of the student samples at each grade level. In addition, kindergarten schools are sampled to be nationally representative; all schools in the following years are representative of the students in the sample attending those schools (rather than being intrinsically nationally representative like the base year). We limit our sample of schools to public schools present in both the Grades 1 and 2 Spring data collection rounds. These grades represent the first period in the dataset in which RTI implementation data is collected from schools, so we choose these grades to avoid additional temporal dependency in the measurement of RTI in grades 3–5. In addition, given the potential increase in the use of RTI over time (or changes in the uses of RTI over time), using data from earlier years (2012–2013) represents an earlier stage in the uptake of RTI practices and thus includes potentially more variability in how/why schools chose to use math RTI.

### Measures

#### Outcome

##### School administrator report of RTI implementation

The outcome of our study is the school administrator’s report of whether their school implemented math RTI in the Spring of 2012–2013 (the Grade 2 round of data collection at the child level). This data were collected as part of the school administrator survey portion of the ECLS-K: 2011. Responses were recorded as “not applicable” (e.g., schools in which RTI would not have been implemented), “no,” “partially implemented,” or “fully implemented.” We collapse partial and full implementation into a single category, resulting in a binary 0 (no)/1 (yes) indicator of RTI. It is unclear what exactly would differentiate “partial” vs. “full” implementation in this survey, so we focus on the presence of any math RTI practices rather than a gradient of implementation (e.g., different forms of partial implementation could exist but there is no way to determine this, which reduces the practical value of differentiating the responses).

#### Predictors

We selected a thorough set of predictors that represented contextual (e.g., economic, community), student (e.g., enrollment, reports of safety issues), personnel factors (e.g., teacher training), and implementation factors (e.g., previous implementation of behavior and writing RTI) to cover the reasonable potential range of reasons schools may adopt math RTI. A sample of the predictors is displayed in Table 1. All predictors are from the Spring semester of Grade 1 and have been aggregated at the school level. The mean was utilized for continuous variables for aggregation purposes, while the mode (we focus on the most frequent category within a school) was employed for categorical variables. The mean was chosen for aggregating continuous variables because it is the most commonly used measure to describe central tendency in continuous data (Clowes and Duke, 2022). This method is also widely utilized in applied research for aggregating such variables (e.g., Geronimus and Bound, 1998; Jacob et al., 2014; Moineddin and Urquia, 2014). For categorical variables, the mode was selected because it is often the most appropriate, and sometimes the only, method to effectively describe the central tendency for nominal variables (Clowes and Duke, 2022). The outliers in our dataset may not pose a problem, as most continuous variables we used are at the school level, meaning that students within the same school share the same value. However, one variable, X4SESL_I, which is the continuous Socioeconomic Status (SES) index, may have potential outliers. Therefore, we checked for outliers in X4SESL_I using Z-scores for each school with at least 10 observations. If a *Z*-score exceeded 3, the observation was identified as an outlier. The results showed that only one school (id = 1,816) had a single outlier, while other schools did not have any outliers.

**Table 1:** Covariates domains considered for RTI prediction.

Covariate block	Covariate description
School demographic factors	School-level sociodemographic factors, including funding mechanisms (Title 1 and 3), poverty, geographic locale (rural, suburban, etc.), changes in enrollment/funding/staffing/class sizes, school-level race/ethnicity, and language status
Policy or procedural features	School policies and procedures for behavioral and academic intervention (e.g., implementation of other tiered systems for behavior and writing, staff procedures for implementing interventions and using data to make decisions, and professional development of staff)
Staffing and administration	General school policies related to staffing and administrative procedures (e.g., academic standards, resource allocation, staffing, school-level procedures for teaching such as using achievement groupings)
Areas of concern for school	Student problems at school (e.g., reports of whether the school has problems with weapons, theft, classroom disorder, and absenteeism)
Community engagement	Community engagement with the school (e.g., before and after-school care, having parent teacher conferences, community support)

### Procedure

Public-use ECLS-K: 2011 data were downloaded from the National Center for Education Statistics website. Data cleaning and analysis took place in Stata 17 (StataCorp, 2021) and R (R Core Team, 2021). To be specific, elastic net logistic regression with nested CV was conducted by using *nestcv* package in R (Lewis et al., 2023). In addition, missing value imputation carried out with the *missForest* package in R (Stekhoven and Bühlmann, 2012; Stekhoven, 2013).

### Data analysis

#### Multiple imputation for missing data

In this study, the percentage of missing values is 16.4%. Little and Rubin (2019) described three missing data mechanisms: Missing Completely at Random (MCAR), Missing at Random (MAR), and Missing Not at Random (MNAR). MCAR situations are characterized by missing values that do not correlate with either observed or unobserved variables. MAR, on the other hand, describes instances where missing values are linked to observed variables but not to unobserved ones. MNAR pertains to cases where missing values are related to unobserved variables. We assume that the missingness present among school-level variables in this dataset is MCAR or MAR given that much of the data comes from administrative information about schools or information that is aggregated to the school level. We would not expect missing data to be related to endogenous, unobserved factors about the schools themselves (MNAR). Rather, we expect that missing responses would be due to random survey nonresponse or non-response related to other systematic factors for which we have information (e.g., other school and teacher characteristics).

Given MAR data, it was necessary to employ an imputation method to address these missing values (Little and Rubin, 2019). We employed the Random Forest (RF) algorithm for multiple imputation. This choice was made because the RF algorithm (1) is well-suited for data missing at random, (2) can effectively handle both continuous and categorical variables, and most importantly, (3) does not require parametric forms and can effectively account for any non-linear relationships, complex interactions, and high dimensionality in the imputation model (Stekhoven and Bühlmann, 2012).

The last advantage of RF is theoretically shared by other nonparametric machine-learning methods. We selected RF based on existing comparisons of RF against other parametric and nonparametric machine-learning imputation methods. Pantanowitz and Marwala (2009) conducted an analysis using empirical data to compare five imputation methods: RF, Autoassociative Neural Network, Autoassociative Adaptive Neuro-Fuzzy Inference System, a hybrid of Random Forest and Autoassociative Neural Network, and a hybrid of Autoassociative Neural Network and Random Forest. Their findings revealed that Random Forest outperforms the other methods in terms of both accuracy and computational efficiency. Similarly, Stekhoven and Bühlmann (2012) explored four different imputation techniques: RF, *k*-Nearest Neighbors algorithm, Missingness Pattern Alternating Imputation and *l*_1_-penalty algorithm, and Multivariate Imputation by Chained Equations, across various empirical datasets. Their findings also revealed that RF had better performance than the other methods generally, particularly in datasets containing both continuous and categorical variables. Tang and Ishwaran (2017) conducted a simulation study to evaluate the performance of various Random Forest (RF) algorithms under three missing data mechanisms. Their findings revealed that RF algorithms generally performed well when data were MCAR or MAR, and the proportion of missing data was low to moderate.

Furthermore, RF managed to maintain acceptable performance in MNAR conditions when the variables were highly correlated (Tang and Ishwaran, 2017). To maximize the information contained in the datasets and to capture possible relationships between missing values and other variables, all variables were used for imputation. This further makes the imputation model robust to account for variables responsible for missingness. Additionally, an independent complete dataset was utilized to evaluate the generalizability of the models, which also served as a sensitivity analysis for missing data imputation.

The RF imputation process was carried out 10 times, resulting in a total of 10 datasets (Musoro et al., 2014; Rubin, 1987; Musoro et al., 2014; Zahid et al., 2020). Generally, the RF imputation employed in this study involves modeling each variable with missing values as a function of all other available variables, with the missing values being predicted using a fitted random forest model. Specifically, an initial guess for the missing values, such as the mean, is made. Variables are then sorted by their percentage of missing values, and the one with the fewest missing values is imputed first. This imputed variable is treated as the response variable, with the others serving as predictors. An RF model is constructed to predict the missing values of this variable, and these missing values are updated by the RF-model-based predicted values. Then, the variable having the next fewest missing data is imputed based on the already imputed variable and others, following the same procedure until the stopping criteria are met. The stopping criteria involve observing an increase in the difference between the newly imputed values and the previously imputed values across observations for the first time, at which point the RF algorithm is stopped. Importantly, this increase should be observed across all types of variables. This non-parametric method, detailed by Stekhoven and Bühlmann (2012), is effective regardless of whether the missing values occur in independent or outcome variables.

When conducting the RF imputation, missing values in the two variables “W4C4P_4TSTR” and “W4C4P_4TPSU” were not imputed, as they represent the strata and primary sampling units (PSU) from the complex survey design. Due to their nature, these variables were not suitable for imputation using the RF algorithm like the other variables. To address missing values of these two variables while retaining as many observations as possible, a value of 0 was assigned to indicate “not specified” for both variables. Although “W4C4P_4TSTR” and “W4C4P_4TPSU” cannot be directly imputed by the RF algorithm, they were included in the RF imputation process to facilitate the imputation of other variables. We chose to include these sampling variables in the RF algorithm because including them in the model allows for control over the sampling design for the ECLS-K dataset (Stapleton and Kang, 2018).

#### Elastic net logistic regression with nested cross-validation under four methods of variable selection

After completing the imputation phase, we conducted elastic net logistic regression analyses using nested CV with 5-fold on each imputed dataset. The elastic net regression was proposed by Zou and Hastie (2005), which is expressed as:


$$\arg\min\left(\frac{1}{2n}\overset{N}{\underset{i=1}{\sum }}{\left(y_{i}-\left[\beta_{0}+\overset{p}{\underset{j=1}{\sum }}\beta_{j}x_{ij}\right]\right)}^{2}+\lambda \left[\frac{\left(1-\alpha \right)}{2}{\left\|\beta \right\|}_{2}^{2}+\alpha {\left\|\beta \right\|}_{1}\right]\right)$$

where ${\left\|\beta \right\|}_{1}=\overset{p}{\underset{j=1}{\sum }}|\beta_{j}|$ and ${\left\|\beta \right\|}_{2}=\sqrt{\overset{p}{\underset{j=1}{\sum }}\beta_{j}^{2}}$ . In our case, $y_{i}=\log\left(\frac{\phi_{i}}{1-\phi_{i}}\right)$ , the logarithm of the odds ratio of RTI implementation to no RTI implementation (or the logit).

Here, ${\left\|\beta \right\|}_{1}$ is the sum of absolute values of the coefficients for the predictors, which is also called $l_{1}$ penalty, and ${\left\|\beta \right\|}_{2}^{2}$ is the sum of squared coefficients for the predictors, which is also called $l_{2}$ penalty. In addition, λ controls the overall strength of regularization, while α balances between LASSO and Ridge regression penalties. When α=1, elastic net regression transforms to LASSO regression, and when α=0, it becomes Ridge regression. The factor 1/2 before the *l*_2_ penalty is included for mathematical optimization convenience and does not alter the fundamental behavior of elastic net regression. Both LASSO and Ridge regressions have limitations with highly correlated predictors: Ridge regression tends to retain both variables but produces similar coefficient estimates for these predictors, whereas LASSO typically selects one predictor and discards the other. However, elastic net regression strikes a balance between LASSO and Ridge. This allows elastic net regression not only to retain both correlated predictors but also to generate stable coefficient estimates. Therefore, elastic net regression is chosen because it combines the advantages of both LASSO and Ridge regressions (Zou and Hastie, 2005).

**Figure 2:**
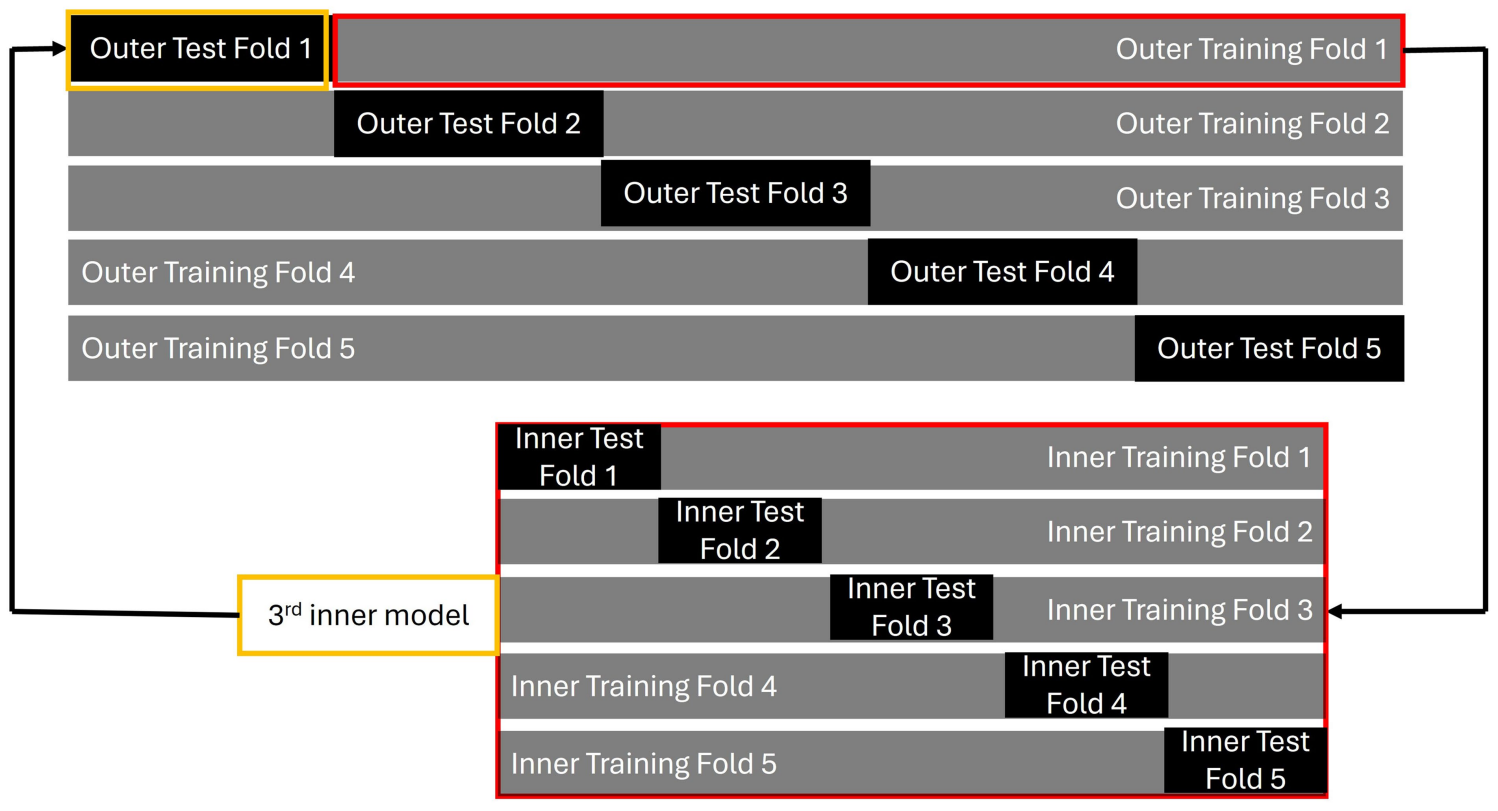
An illustration of nested CV.

Elastic net regression selects variables by imposing two penalties on variable coefficients; therefore, choosing the appropriate values for λ and α is crucial. In this study, these penalty parameters were chosen via nested CV because Vabalas et al. (2019) have demonstrated that nested CV is an effective cross-validation method, especially when the sample size is small. The procedure for nested CV is presented in Figure 2. A 5-fold nested CV is used in this study as an example. The standard nested CV is conducted in six steps:

1. The entire dataset is divided into several folds; for example, into 5-fold. These serve as the outer folds of the nested CV. Each outer fold is further split into an outer testing set and an outer training set.
2. Each outer training set is further divided into several inner folds. These inner folds consist of their own training and testing sets, referred to as the inner training fold and inner testing fold, respectively. These are used for feature selection, hyperparameter tuning, and model building. The best model for each inner fold is selected based on the smallest discrepancy in performance between the inner training and testing sets, indicating minimal overfitting.
3. The best model from an inner fold (for example, the model from the third inner fold, highlighted in yellow) is then tested using the corresponding outer testing set (in this case, outer test fold 1, also highlighted in yellow).
4. Steps 2 and 3 are repeated for each outer fold, with each outer fold producing a model.
5. The best features and tuning parameters are chosen from the model associated with the outer fold that shows the least overfitting. These are then used to train a model on the entire dataset to create the final model.
6. The final model is applied to an independent dataset to validate its generalizability.

All predictors were standardized before conducting elastic net logistic regression. While elastic net logistic regression offers benefits, it can potentially yield 10 distinct models corresponding to the 10 imputation datasets. This diversity presents a challenge in synthesizing an overarching model from the 10 distinct versions. Consequently, upon obtaining these 10 models, we employed four distinct methods to select the final candidate models, resulting in four separate candidate models. Each method yields its own model for consideration in the final selection process. Four methods of selecting the candidate models were comprehensively described in the next section. To determine the final model, the candidate models were subsequently assessed in an independent dataset to evaluate their generalizability. All candidate models used predictors from the independent dataset to generate predictions for math RTI. Subsequently, the balanced accuracy between these predicted math RTI values and the observed math RTI from the independent dataset was calculated. The balanced accuracy is calculated as $\left(\mathrm{sensitivity}+\mathrm{specificity}\right)/2$ , where in our case sensitivity is the rate of correctly labeling schools as implementing RTI and specificity is the rate of correctly labeling schools as not implementing RTI (Chicco et al., 2021; García et al., 2009).

Because the predicted math RTI from the model is expressed as a percentage, we utilized the optimal threshold to categorize the predicted math RTI into two distinct categories. The optimal threshold applied in this study is widely recognized in research for maximizing both sensitivity and specificity (Coffin and Sukhatme, 1997; Perkins and Schisterman, 2006; Unal, 2017). This approach is also called the “closest-top-left-corner” method. This method utilizes the receiver operating characteristic (ROC) plot, a graphical representation that maps the True Positive Rate (*Sensitivity*) against the False Positive Rate (1—*Specificity*) for various threshold values. Typically, the *x*-axis denotes 1-*Specificity*, while the *y*-axis corresponds to *Sensitivity*. Ideally, the best classifier would achieve 100% Sensitivity and 0% False Positive Rate, which would be represented by the point (0, 1) on the ROC plot, situated at the top-left corner of the graph. However, attaining this perfect point is nearly impossible in practical scenarios. Therefore, the “closest-top-left-corner” method selects the point on the ROC curve that is nearest to (0, 1) as the optimal threshold, representing the most effective balance between *Sensitivity* and *Specificity* based on the available data. Beyond determining the optimal threshold, the ROC plot is also utilized to calculate the Area Under the Curve (AUC), which quantifies the area beneath the ROC curve. An AUC of 1 indicates that the elastic net logistic regression model is an ideal classifier, perfectly distinguishing schools by their math RTI status. Conversely, an AUC of 0.5 suggests that the model lacks discriminative ability (Fan et al., 2006; Obuchowski and Bullen, 2018). The final model which exhibited the best performance was chosen.^1^

Considering the necessity for an independent dataset to assess generalizability, we divided the dataset into two parts: one for model building, which includes training and validating the model, and another to serve as an independent dataset for conducting the generalizability check of the model. In addition, to ensure the validity of the generalizability assessment, we employed complete cases from the original dataset, which were free from missing values, to serve as the independent dataset. This independent dataset consisted of 127 schools. The rest of the cases were used for training and validating the elastic net logistic regression model.

Given the prevalence of Likert scale items in our data, we faced the challenge of potentially expanding the number of predictors significantly if each were to be treated categorically. To address this and streamline the modeling process, we adopted the guidance provided by Harpe (2015), treating ordinal variables with five or more categories as continuous, while those with four or fewer categories were handled as categorical variables. This decision was made to balance the granularity of the Likert-scale responses with the practical considerations of model complexity and interpretability.

The second consideration is related to the sampling design. Again, we applied the same strategy to control for the sampling design as we did in the missing value imputation stage. We included “W4C4P_4TSTR” and “W4C4P_4TPSU,” representing strata and PSU, to account for the sampling design. Consequently, these two variables were included in the elastic net logistic regression analysis without undergoing variable selection. The variables “W4C4P_4TSTR” and “W4C4P_4TPSU” are inherently categorical and would typically necessitate the creation of dummy variables. Upon thorough examination of the cross-tabulations between “W4C4P_4TSTR” and the response variable “math RTI,” as well as “W4C4P_4TPSU” and “math RTI,” we identified many cells with zero observations. The details of these cross-tabulations are presented in Tables 2, 3. Such a distribution poses a risk of the complete separation or quasi-complete separation problem in logistic regression (Devika et al., 2016). To mitigate this issue, we strategically combined certain categories within “W4C4P_4TSTR” and “W4C4P_4TPSU.” This not only resolved the separation problem but also curbed the risk of overfitting in the final model.

**Table 2:** Cross-tabulation between “W4C4P_4TSTR” and “math RTI” based on school level.

		W4C4P_4TSTR												
		0	1	2	3	4	5	6	7	8	9	10	11	12
Math RTI	0	34	0	0	0	1	0	1	0	3	4	1	0	0
	1	185	7	1	3	1	7	11	14	15	4	6	8	2
		W4C4P_4TSTR												
		14	15	16	19	20	21	22	23	24	26	28	29	33
Math RTI	0	1	0	0	0	0	0	0	0	0	0	0	0	0
	1	8	4	2	1	3	1	1	2	2	2	1	1	2
		W4C4P_4TSTR												
		35	36	37	38	39	40	41	42	43	44	45	46	47
Math RTI	0	2	1	7	0	0	1	0	0	4	1	5	2	2
	1	10	15	9	15	14	6	16	10	16	8	16	10	13
		W4C4P_4TSTR												
		48	49	50	51	52	53	54	55	56	57	58	59	60
Math RTI	0	2	0	1	2	0	2	1	1	1	2	0	1	2
	1	17	19	18	30	19	21	14	24	8	12	10	13	7
		W4C4P_4TSTR												
		61	62	63	64	65	66	67	68	69				
Math RTI	0	0	0	1	6	0	0	0	0	1				
	1	6	20	7	14	9	8	16	14	21				

W4C4P_4TSTR: sampling strata; Math RTI: whether math RTI is implemented.

**Table 3:** Cross-tabulation between “W4C4P_4TPSU” and “math RTI” based on school level.

		W4C4P_4TPSU								
		0	1	2	3	4	5	6	7	8
Math RTI	0	34	24	24	3	0	1	2	2	0
	1	185	221	262	60	12	6	11	6	4
		W4C4P_4TPSU								
		9	10	15	18	19				
Math RTI	0	2	0	1	0	0				
	1	5	4	0	1	2				

W4C4P_4TPSU: sampling PSU; Math RTI: whether math RTI is implemented.

Based on these two cross-tabulations, the re-categorization of “W4C4P_4TSTR” and “W4C4P_4TPSU” is primarily determined by the category of the response variable where math RTI equals 0, given the majority of zero observations occur when math RTI is 0. Additionally, the recategorization was implemented to balance the sample sizes between the math RTI = 0 group and the math RTI = 1 group. This recategorization of “W4C4P_4TSTR” and “W4C4P_4TPSU” was conducted at the school level. For “W4C4P_4TSTR,” excluding those with missing values (NAs), schools were categorized based on the math RTI sample size. Schools with math RTI cell values of 14 or fewer were grouped together into the first category and assigned a code of 1. Schools with math RTI cell values in the range [15, 20) were grouped into the second category and coded as 2, and those with math RTI cell values are equal to or greater than 20 were grouped into the third category and coded as 3. In addition, those who have NAs were coded as 0. Regarding “W4C4P_4TPSU,” except for those who have NAs, the original categories 1 and 2 were retained due to the substantial number of values present in the math RTI cell, as they provided a sufficient sample size for both the math RTI = 0 group and the math RTI = 1 group. The rest of the categories were combined together and coded as 3. Also, those who have NAs were coded as 0.

It is important to note that this category consolidation was uniquely applied to “W4C4P_4TSTR” and “W4C4P_4TPSU.” The rationale behind this selective approach is twofold: First, other categorical variables were subjected to the selection process of the elastic net logistic regression, which inherently manages the separation problem (Friedman et al., 2010; Münch et al., 2021). Second, “W4C4P_4TSTR” (49 categories) and “W4C4P_4TPSU” (12 categories) contain too many categories. After dummy coding these variables, the number of predictors increases dramatically, potentially leading to overfitting in the final model. This issue arises because “W4C4P_4TSTR” and “W4C4P_4TPSU” are integral to accounting for the sampling design and were retained without penalties. Moreover, these two variables were not the central focus of our study, further justifying their fixed inclusion in the model without the application of elastic net penalties.

#### Four methods of aggregating results from multiple imputation

As previously discussed, the process could yield 10 distinct models, each corresponding to one of the 10 imputation datasets. Consequently, the crucial challenge lies in determining the candidate models for the final selection, especially considering that these models may contain feature-varying variables. Some studies have discussed the variable selection of regularized linear regression (Musoro et al., 2014; Gunn et al., 2023; Zahid et al., 2020). Although the studies by Gunn et al. (2023), Zahid et al. (2020), and Musoro et al. (2014) primarily utilized LASSO regression, the methodologies they explored can be adaptable to elastic net regression. Consequently, we extended their approaches to our elastic net framework, corresponding to Method_50_, Method_coef_, and Method_adj_, respectively. Furthermore, we proposed a new method to select the candidate model from 10 distinct models based on the generalizability (Method_gen_).

##### Method_50_

Method_50_ initially performs elastic net logistic regression separately on each imputed dataset, retaining variables selected in more than 50% of the cases. The variables “W4C4P_4TSTR” and “W4C4P_4TPSU” were not selected because they were consistently retained in the model. Average the coefficients of the selected variables across the 10 models derived from imputed data to construct the final candidate model. Then, the final candidate model was fitted to independent data to evaluate its generalizability. This method was selected based on empirical evidence from Gunn et al. (2023), which demonstrated its superior performance compared to two other variable selection methods. The first alternative method involves using stacked datasets, where training and testing datasets from multiple imputed datasets are combined into a single stacked training dataset and a corresponding stacked testing dataset. A regularized regression model is then developed using the stacked training dataset and validated using the stacked testing dataset. The second method employs a group penalty approach,^2^ where the group penalty parameter is calculated jointly across the training datasets for each imputed dataset. This approach ensures that models derived from multiple imputed datasets incorporate consistent variables, with each model’s performance evaluated against its respective testing dataset before aggregating the final performance metrics across all models.

##### Method_coef_

Method_coef_, proposed by Zahid et al. (2020), begins by independently conducting elastic net logistic regression on each imputed dataset. It retains variables that meet specific criteria of 1/p , where p is the number of predictors. Notably, the variables “W4C4P_4TSTR” and “W4C4P_4TPSU” were excluded because they were consistently retained in the model. To be more specific, the criteria for choosing variables are:

1. For continuous variable $X_{j}$ associated with a parameter $\beta_{j,m}$ , indicating the *j*th ( j=1,…,p ) predictor within the *m*th ( m=1,…,M ) imputed dataset. The criterion for retaining the variable is as follows:

$$\frac{\frac{1}{M}\sum_{m=1}^{M}|{\hat{\beta }}_{j,m}|}{\sum_{j=1}^{p}\frac{1}{M}\sum_{m=1}^{M}|{\hat{\beta }}_{j,m}|}\geq \frac{1}{p}$$

2. For a categorical variable $X_{j}$ with K+1 categories, K dummy variables are required. Each dummy variable is associated with a parameter $\beta_{jk,m}$ , which represents the *k*th ( $k=1,\ldots ,K_{j}$ ) dummy variable for the *j*th ( j=1,…,p ) predictor within the *m*th ( m=1,…,M ) imputed dataset. The criterion for retaining the variable is as follows:

$$\frac{\frac{1}{M\cdot K_{j}}\sum_{k=1}^{K_{j}}\sum_{m=1}^{M}{\hat{|\beta }}_{jk,m}|}{\sum_{j=1}^{p}\frac{1}{M\cdot K_{j}}\sum_{k=1}^{K_{j}}\sum_{m=1}^{M}|{\hat{\beta }}_{jk,m}|}\geq \frac{1}{p}$$

Then, average the coefficients of the selected variables across the 10 models derived from imputed data, and fit the final candidate model to independent data to evaluate its generalizability.

Zahid et al. (2020) explored the influence of the number of predictors ( 40,80,200,500 ) and proportions of missingness ( 0.05,0.1,0.2,0.3 ) on Method_coef_ with a small sample size ( 100 ). The results revealed that Method_coef_ can relatively balance the trade-off between selecting relevant and irrelevant variables. Typically, selecting more relevant variables tends to also increase the selection of irrelevant variables. In addition, Method_coef_ can be used when the number of predictors exceeds the sample size, a scenario where the method using group penalty fails to select variables.

##### Method_adj_

Method_adj_, proposed by Musoro et al. (2014), differs from previous methods. While the earlier methods finalize the model through variable selection, this method does not engage in selecting variables. Instead, its aim is to adjust the parameter estimates of the final model. The procedure for Method_adj_ is outlined as follows:

1. Run elastic net logistic regression independently for each imputed dataset.
2. Disregard the variable distinctions among each model and compute the average coefficients for all parameters across the 10 models, ${\hat{Y}}_{\mathrm{fin}}=\hat{\alpha }+{\hat{\beta }}_{j}X_{j}$ , where $\beta_{j}$ is the coefficient for the predictor $j\left(j=1,\ldots ,p\right)$ .
3. Use bootstrapping to obtain the calibration parameters for the parameter adjustment of the final model. Here is the procedure of bootstrapping:

  For a bootstrap run, the same observations were selected across 10 imputed datasets ( $imp_{i}$ , where i=1,…,10 ) to obtain corresponding bootstrapping datasets, $imp_{i}^{*}$ ( i=1,…,10 ).
  Rebuild the elastic net logistic regression model based on the bootstrapping datasets, $imp_{i}^{*}$ .
  Repeat a and b 100 times.

4. Aggregate each variable in the imputed datasets into a long-stacked dataset, so that the size of the stacked dataset is 10 times that of the original dataset. For each bootstrap run, the predicted response variable is calculated using the coefficients derived from the elastic net regression model built on the bootstrap sample. Specifically, the predicted response variable is computed by multiplying the elastic net regression coefficients with the predictors in the stacked dataset. For predictors not selected by the elastic net regression, their coefficients are set to zero. Subsequently, calibration parameters are computed by regressing the response variable in the stacked dataset against the predicted response variable. The predicted response variable from bootstrapping is calculated using the final model from step 3 and predictors from the stacked dataset. This relationship is symbolized as $Y_{\mathrm{imp}}=\alpha_{adj}+\beta_{adj}{\hat{Y}}_{imp^{*}}$ .
5. Then, compute the average of $\alpha_{adj}$ and $\beta_{adj}$ over these 100 bootstrap iterations to obtain ${\bar{\alpha }}_{adj}$ and ${\bar{\beta }}_{adj}$ . Finally, adjust the final model for intercepts and all coefficients from the imputed datasets by using the equation ${\bar{\alpha }}_{adj}+{\bar{\beta }}_{adj}\left(\hat{\alpha }+{\hat{\beta }}_{j}X_{j}\right)$ .
6. Fit the final model to the independent data to assess the generalizability.

##### Method_gen_

The final method, Method_gen_, is a novel approach introduced by this study. Method_gen_ initially conducts elastic net logistic regression independently on each imputed dataset. Each model is then fitted to independent data, with the final candidate model being selected based on achieving the highest balanced accuracy.

#### Evaluation criteria for the model performance

Given that the response variable math RTI is binary, accuracy serves as a suitable metric to assess the model performance. However, the response variable, math RTI, in this study is imbalanced. Sun et al. (2009) claimed that standard classifiers, such as the logistic regression and decision tree, tended to ignore the rare cases, potentially compromising the accuracy of the model’s predictions. Given the presence of an imbalanced response variable in the dataset, weights were computed for each observation. These weights were then applied to the regularized logistic regression to ensure a balanced 50:50 weight ratio across the two categories. Also, as we focus on the prediction accuracy for both implementing and not implementing RTI, balanced accuracy is a more precise metric as compared to regular accuracy. Furthermore, we also used the receiver operating characteristic (ROC) plot and the corresponding area under the curve (AUC) to delineate the performance of the elastic net logistic regression.

## Results

### Descriptive statistics

The total sample size at the individual level was 6,647. After aggregating variables to the school level, the sample size was reduced to 1,130. Since math RTI was investigated at the school level, the final sample size should be 1,130. Within the selected data, there were 37 continuous predictors and 54 categorical ones. Upon creating dummy variables for categorical variables, the analysis included a total of 160 predictors. Across the 10 imputed datasets, the count of cases where math RTI was not implemented ranged from 94 to 97. Conversely, for cases where math RTI was implemented, the count lied between 1,033 and 1,036. This data suggest that the majority of schools have adopted math RTI. However, due to the large number of predictors, descriptive statistics of predictors were omitted.

### Four methods of variable selection and the final model

Table 4 displays the candidate models derived from four methods. Except for variables of the sampling design, W4C4P_4TSTR and W4C4P_4TPSU, we found Method_gen_, Method_50_, and Method_coef_ selected the same variables. Method_adj_ does not select variables from the 10 models derived from imputed data. Instead, it retains all variables from these 10 models and adjusts their coefficients. As a result, Method_adj_ includes all variables produced by the imputed data, resulting in a slightly larger variable count.

**Table 4:** Candidate models from four methods.

Methods	Variables
Method_gen_	W4C4P_4TSTR.1, W4C4P_4TSTR.2, W4C4P_4TSTR.3, W4C4P_4TPSU.1, W4C4P_4TPSU.2, W4C4P_4TPSU.3, S4RTLMTH.2
Method_50_	W4C4P_4TSTR.1, W4C4P_4TSTR.2, W4C4P_4TSTR.3, W4C4P_4TPSU.1, W4C4P_4TPSU.2, W4C4P_4TPSU.3, S4RTLMTH.2
Method_coef_	W4C4P_4TSTR.1, W4C4P_4TSTR.2, W4C4P_4TSTR.3, W4C4P_4TPSU.1, W4C4P_4TPSU.2, W4C4P_4TPSU.3, S4RTLMTH.2
Method_adj_	W4C4P_4TSTR.1, W4C4P_4TSTR.2, W4C4P_4TSTR.3, W4C4P_4TPSU.1, W4C4P_4TPSU.2, W4C4P_4TPSU.3, S4RTLMTH.2, S4RTLSOC.2

Variables beginning with S4 are administrator-reported; variables beginning with A4 are teacher-reported; variables beginning with X4 are data collected by/reported to the ECLS-K. W4C4P_4TSTR: sampling strata, W4C4P_4TPSU: sampling PSU, S4RTLMTH: whether math RTI is implemented in Grade 1, S4RTLSOC: whether behavior/social RTI is implemented in Grade 1. Variables with numbers indicate those are dummy variables for corresponding categorical variables. For dummy variables with “RTL” (which indicates the variable measuring administrator-reported RTI implementation), the baseline represents full implementation. A value of “2” denotes no implementation, while “1” is partial implementation.

**Figure 3:**
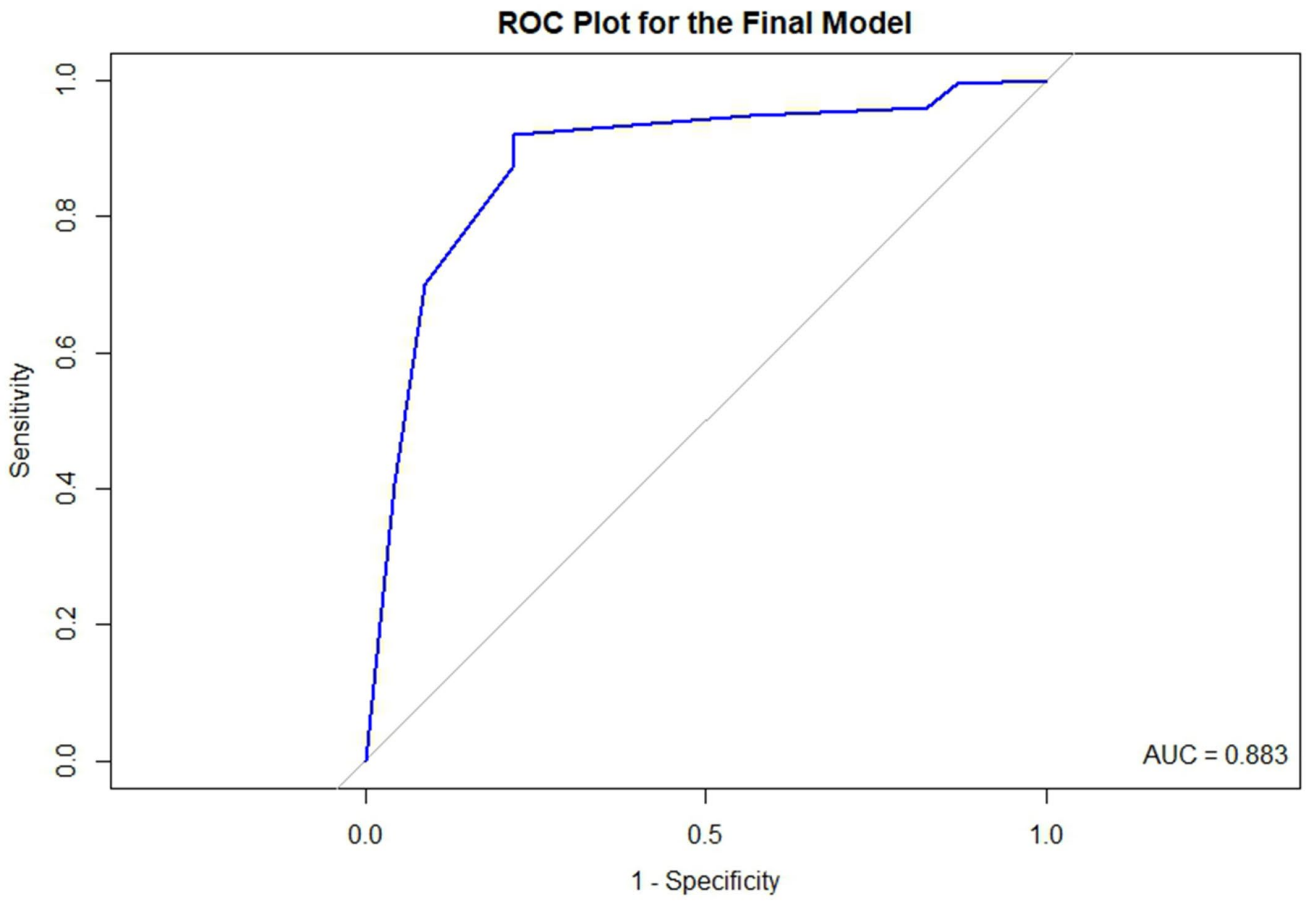
The ROC plot for the final model.

Table 5 presents the balanced accuracy and AUC values associated with the four methods. Method_50_ and Method_coef_ achieved the highest balanced accuracy, both equal to 0.852, and also demonstrated the highest AUC values. In contrast, Method_gen_ also showed commendable balanced accuracy at 0.829, while Method_adj_ recorded the lowest balanced accuracy of 0.491 among the four methods. Given the superior performance of Method_50_ and Method_coef_, the final model is based on these methods, as they produced identical results. Therefore, the final model is defined by the following logistic regression model: $\begin{array}{l} \log\left(\frac{P\left(Y=1\right)}{1-P\left(Y=1\right)}\right)=.186-.038\times W4C4P\_4\mathrm{TPSU}.1+.166\times W4C4P\_4\mathrm{TPSU}.2\\ -.310\times W4CP\_4\mathrm{TPSU}.3+.799\times W4C4P\_4\mathrm{TSTR}.1+.112\times W4C4P\_4\mathrm{TSTR}.2\\ -.102\times W4C4P\_4\mathrm{TSTR}.3-1.072\times S4\mathrm{RTLMTH}.2\text{.} \end{array}$ Figure 3 is the ROC plot for the final model derived from Method_50_ and Method_coef_. Based on the balanced accuracy and the ROC plot, it is concluded that the final model effectively classifies math RTI. Table 6 presents the tuning parameters for the 10 imputed datasets.

**Table 5:** Generalizability of candidate models from four methods using the independent dataset.

	Balanced accuracy	AUC
Method_gen_	0.829	0.855
Method_50_	0.852	0.883
Method_coef_	0.852	0.883
Method_adj_	0.491	0.329

**Table 6:** Tuning parameters of 10 elastic net logistic regression models.

	λ	α
Imputed dataset 1	0.235	0.500
Imputed dataset 2	0.102	0.800
Imputed dataset 3	0.136	1.00
Imputed dataset 4	0.160	1.00
Imputed dataset 5	0.109	0.600
Imputed dataset 6	0.133	1.00
Imputed dataset 7	0.131	1.00
Imputed dataset 8	0.133	1.00
Imputed dataset 9	0.137	0.600
Imputed dataset 10	0.135	0.800

## Discussion

Response to intervention has been prominent in research for several decades, and since the early 2000s, it has become much more prominent in practice both through individual districts’ uptake of the practice as well as through recommendations in federal and state policies (Jimerson et al., 2016). The overall process, typically involving a three-tiered system to classify the intensity of students’ academic needs, involves many moving components, including implementation of assessment practices like screening and progress monitoring, managing data-based decisions based on screening and monitoring data, and selecting appropriate curricula and interventions to implement at each tier. Each component of RTI requires substantial human, material, and financial resources, and there is substantial between-school variability in the capacity to allocate resources and sustain implementation. However, little is known about the factors that relate to schools’ decisions to use and sustain RTI, though it is reasonable to assume economic and human resources would be a primary driver.

In this study, we investigated the predictors of schools’ implementation of math RTI in Grade 2 using predictors from the prior year. There is a dearth of research examining the factors that relate to schools’ decision to use RTI in math. Historically, large-scale studies of reading RTI and its implementation have predominated the literature (e.g., Balu et al., 2015), and the decisions to implement math RTI in addition to other initiatives in place (Mason et al., 2019) may be unique relative to decisions guiding implementation of other prevention and intervention systems. Thus, it is important to establish an empirical basis for the factors that relate to schools’ uptake of an initiative like math RTI, which is both complex in terms of the school system dynamics needed to sustain its implementation and resource-demanding (Choi et al., 2022; Mason et al., 2019).

Results of our analyses indicate that three of the four methods evaluated for selecting predictors were equivalent in model balanced accuracy. Of these three methods, Method_50_ and Method_coef_ equally demonstrated the strongest performance based on the AUC and balanced accuracy. All models demonstrated that previous math RTI implementation was a predictor of future math RTI implementation. Additionally, RTI implementation for social/behavioral skills in Grade 1 emerged as a potential predictor for math RTI implementation, as it was included in several elastic net regression models. This finding is further supported by the correlation analysis, which showed that RTI implementation for math and social/behavioral skills in Grade 1 were the top two variables most strongly correlated with math RTI implementation in Grade 2. Specifically, the correlation for math RTI implementation in Grade 1 was 0.602, while the correlation for RTI implementation for social/behavioral skills in Grade 1 was 0.224. Notably, RTI implementation for math in Grade 1 exhibited a significantly higher correlation with math RTI implementation in Grade 2 compared to other variables. This could suggest that concurrent RTI infrastructure may be a determinant of math RTI implementation, though this may not be as robust of a predictor given the inconsistency of its selection into the models. Given that this is the first study to employ these methods for predicting RTI implementation, it is essential to conduct additional research examining the school-level and contextual factors relating to schools’ math RTI implementation decisions. Qualitative reports of math RTI implementation indicate student economic conditions, teacher professional development, and other implementation priorities are relevant factors in the math RTI implementation process (Mason et al., 2019).

### Different methods of variable selection

In this paper, we used four methods, Method_gen_, Method_50_, Method_coef_, and Method_adj_, to select candidate models from 10 distinct models derived from 10 imputed datasets. Then, four candidate models were evaluated in the independent dataset and the final model was determined based on the best performance. From Tables 4, 5, we could observe that Method_50_, and Method_coef_ selected the same variables and exhibited identical balanced accuracy and AUC in the independent dataset. This consistency is due to the robustness of the RF imputation method used in our study against multiple imputations. To further confirm this robustness, we generated an additional 30 imputed datasets, bringing the total to 40. Among these, only two yielded elastic net regressions with different variables, while the remainder produced identical elastic net regressions, albeit with varying parameter estimates. This consistency ensures that both Method_50_ and Method_coef_ select the same variables across multiple imputations. Moreover, the variable S4RTLMTH.2, which is included in the final model, consistently appears in all elastic net models. Consequently, the average coefficient for S4RTLMTH.2 was calculated across the multiple imputed datasets, resulting in the same final model being produced by both Method_50_ and Method_coef_.

In contrast, although Method_gen_ selected the same variables as Method_50_ and Method_coef_, it achieved different balanced accuracy and AUC on independent data. This variation arises because Method_gen_ selects only one model from the candidate models based solely on AUC, without averaging the coefficients of the selected variables across these models. Consequently, the parameter estimates in the final model of Method_gen_ differ from those in Method_50_ and Method_coef_. In scenarios where the outcome is imbalanced, the AUC can provide an overly optimistic view of a model’s performance, particularly favoring the majority class. Therefore, when the outcome is imbalanced, balanced accuracy is a more appropriate index than AUC, because balanced accuracy takes this imbalance into consideration. Given the similar performance observed with Method_gen_, Method_50_, and Method_coef_, researchers are encouraged to employ all these approaches in future studies. Doing so can allow these methods to complement one another, providing a more comprehensive understanding of the study. However, Method_adj_ is not recommended as it underperformed compared to the other three methods and potentially includes too many variables, which may lead to overfitting.

Method_adj_ is different from the other three methods because it retains all variables from 10 distinct models derived from 10 imputed datasets and adjusted model coefficients rather than selecting variables. This retention of all variables can result in an overly complex final model, prone to overfitting. The relatively poor performance of Method_adj_ may be attributed to its approach of calculating the calibration parameters based on the stacked dataset of these 10 imputed datasets. The 10 imputed datasets were used both for constructing the elastic net logistic regression model and for computing the calibration parameters. This repeated use of the same datasets may cause model overfitting, which could degrade performance and compromise generalizability. Another potential reason for Method_adj_’s poor performance might be that the bootstrapping datasets do not accurately represent the original dataset, due to the nature of bootstrapping involving repeated sampling from the original dataset with replacement. Given the significant imbalance in the outcome, the class distributions in the bootstrapping data could differ markedly. Moreover, since the RF imputation produced relatively stable imputed datasets, and Method_adj_ consistently selected the same observations across these imputed datasets for calibration in each run, this approach could cause all bootstrapping datasets in each run to differ significantly in outcome categories from the original dataset, potentially biasing the calibration parameters.

When selecting variables for regularized models with multiple imputations, there are generally two approaches to variable selection. The first involves fitting regularized models separately for each imputed dataset, which may result in distinct models, and then applying thresholds to select variables. The second approach aims to create a unified set of variables across all regularized models. Method_50_ and Method_coef_ adhere to the first approach. Initially, each method fits a regularized model separately for each imputed dataset. Variables are then selected based on specific thresholds: for Method_50_, variables with non-zero coefficients must appear in more than 50% of the cases, whereas for Method_coef_, the magnitude of the coefficients must be equal to or greater than 1/p . The second approach can be achieved either by using a stacked dataset or by applying a group penalty, as described by Gunn et al. (2023) in their second and third methods. As previously mentioned, the stacked method combines multiple imputed datasets into a single stacked dataset, then applies regularized regression to this unified dataset. This approach can select unified variables because it ultimately chooses variables from one dataset. On the other hand, the method using a group penalty applies the group penalty across all imputed datasets, assuming that if a variable is important, it should be selected in all imputed datasets. This method produces unified variables by jointly fitting the group penalty to all imputed datasets. By adopting these two methods, researchers can bypass the need to select a threshold when formulating the final model.

No single variable selection method consistently outperforms others. Previous studies, such as Wood et al. (2008), have shown that the performance of methods using thresholds and stacked datasets is comparable. Du et al. (2022) favored the method using a stacked dataset over the group penalty method for achieving better coefficient estimates and reduced computation time. Conversely, Gunn et al. (2023) observed that the stacked dataset method underperformed compared to methods using thresholds and the group penalty in an empirical dataset. Zahid et al. (2020) noted that while the method using the group penalty can correctly identify relevant variables, it also tends to select more non-informative variables. Moreover, this method fails to select variables when the number of predictors exceeds the sample size. Additionally, the efficacy of the method using the group penalty is highly dependent on the number of imputations. Du et al. (2022) made similar observations regarding the dependency on the number of imputations for the group penalty method, noting a more significant improvement with this approach compared to the stacked dataset method as the number of imputations increased. Previous studies have not reached a consensus on which method is definitively superior. Therefore, researchers are encouraged to employ multiple variable selection methods to assess the robustness of the selected variables.

### Practical implications

The current results confirm practical assumptions that existing initiatives (previous social/behavioral RTI implementation) relate to RTI implementation. Although the first study to empirically demonstrate these relations, these findings are likely unsurprising to applied researchers and school personnel. The capacity and motives to initiate math RTI implementation will be constrained by other existing priorities and competing resources for an added initiative (Mason et al., 2019). However, it is important to note that our results suggest that previous math RTI implementation is consistently selected as a predictor, suggesting strong stability in schools’ decisions to use math RTI over these two school years. In the absence of contextual and human resources predictors, it may be that initial uptake of RTI is a more idiosyncratic process rather than systematically attributable to specific, quantifiable factors. As a result, research and practice would benefit from additional mixed methods work to understand the experiential processes involved in RTI implementation and whether this relates to other systematic factors at the school level.

### Limitations and future directions

One limitation of this study is the small size of the independent dataset. The limited sample size means that some categories of categorical variables cannot be validated in the independent dataset, as it lacks representation of those categories. For example, there were three categories of W4C4P_4TPSU when constructing the elastic net logistic regression model. However, in the independent dataset, only one category of W4C4P_4TPSU was present. Therefore, limited sample size of the independent dataset compromises the generalizability of the final model to some degree. The second limitation of this study lies in our comparison of four methods using empirical data. While the empirical results offer valuable insights, a thorough simulation study is necessary to comprehensively evaluate the four variable selection methods. Moreover, the finding of robustness of the RF imputation is also based on empirical data. A simulation study is needed to fully investigate the relationship between the RF imputation method and the number of imputations. Last, the reports of math RTI implementation in Grade 2, which we further collapsed into 0 = no implementation or 1 = partial/full implementation are highly limited and may not accurately represent the presence of core components of math RTI implementation (Lembke et al., 2012). As a result, the nature of these schools implementing math RTI is unclear. More accurate criteria for differentiating math RTI implementation is essential in future studies to accurately capture the factors that go into schools’ uptake and implementation sustainment. Moreover, the use of school-level data in this case may not accurately represent actual school-level factors: teachers reports are not representative of all teachers within each school, nor are aggregated student-level data representative of all students in that grade and school. Finally, the current study cannot differentiate how “partial” and “full” RTI would have been interpreted. Future research should examine the predictors of different degrees of implementation in addition to the specific processes that are implemented.

## Conclusion

Given the increasing uptake of tiered intervention systems in schools (Choi et al., 2022), such as RTI, there is a pressing need to identify the factors relating to schools’ implementation decisions. Our current study revealed that existing RTI systems were primary predictors of schools’ implementation. This is a first step in developing an empirical basis for predictors of school-wide math RTI implementation.

## Data Availability

The datasets and R code are available in online repositories. The names of the repository/repositories and accession number(s) can be found at: https://www.openicpsr.org/openicpsr/project/209149/version/V2/view.

## Footnotes

^1^The final model was selected based on having the highest balanced accuracy. In cases where two candidate models exhibit identical balanced accuracy, the model with the larger Area Under the Curve (AUC) was chosen.

^2^The calculation of group penalty parameter, λ , as jointly determined using all training datasets (Chen and Wang, 2013) is $\min\overset{M}{\underset{m=1}{\sum }}\overset{N}{\underset{i=1}{\sum }}{\left(y_{mi}-\left[\beta_{m0}+\overset{p}{\underset{j=1}{\sum }}\beta_{mj}x_{mij}\right]\right)}^{2}+\lambda \overset{p}{\underset{j=1}{\sum }}\sqrt{\overset{M}{\underset{m=1}{\sum }}\beta_{mj}^{2}}$ where M is the number of imputed datasets, N is the sample size, p is the number of predictors.
